# Arbuscular Mycorrhizal Fungi as a Salt Bioaccumulation Mechanism for the Establishment of a Neotropical Halophytic Fern in Saline Soils

**DOI:** 10.3390/microorganisms12122587

**Published:** 2024-12-13

**Authors:** Mónica A. Lugo, María A. Negritto, Esteban M. Crespo, Hebe J. Iriarte, Samuel Núñez, Luisa F. Espinosa, Marcela C. Pagano

**Affiliations:** 1 Laboratorio de Micología, Diversidad e Interacciones Fúngicas (MICODIF), Área Ecología, Facultad de Química, Bioquímica y Farmacia, Universidad Nacional de San Luis (UNSL), Ejército de los Andes 950, Bloque I, 2do Piso, Box 4, San Luis 5700, Argentina; ecrespo@email.unsl.edu.ar (E.M.C.); hjiriarte@email.unsl.edu.ar (H.J.I.); 2 Instituto de Investigaciones Biológicas (IMIBIO-CCT SL), Consejo Nacional de Investigaciones Científicas y Tecnológicas (CONICET), Universidad Nacional de San Luis (UNSL), San Luis 5700, Argentina; 3 Grupo de Investigación en Manejo y Conservación de Fauna, Flora y Ecosistemas Estratégicos Neotropicales MIKU, Universidad del Magdalena, Calle 29H3 No. 22-01, Sector San Pedro Alejandrino, Santa Marta 470004, Colombia; mnegritto@unimagdalena.edu.co (M.A.N.); samuelnunez@unimagdalena.edu.co (S.N.); 4 Laboratorio de Microscopía Electrónica de Barrido y Microanálisis (LABMEM), Bloque I, PB, Universidad Nacional de San Luis (UNSL), Ejército de los Andes 950, San Luis 5700, Argentina; 5 Laboratorio de Calidad Ambiental Marina (LABCAM) del Instituto de Investigaciones Marinas y Costeras (INVEMAR), Santa Marta 470006, Colombia; luisa.espinosa@invemar.org.co; 6 Universidade Federal de Minas Gerais, Avenida Antônio Carlos 6627, Pampulha, Belo Horizonte 31270-901, MG, Brazil

**Keywords:** thermal ponds, halophytic fern, arbuscular mycorrhizae, *Glomus*, Glomeromycota, coastal environments, mangroves, Neotropics, plant invasions

## Abstract

*Acrostichum aureum* is a halophytic pantropical invasive fern growing in mangroves and swamps. Its association with arbuscular mycorrhizal fungi (AMF) has been reported in Asia. AMF and their symbiosis (AM) commonly colonise the absorption organs of terrestrial plants worldwide. Furthermore, AMF/AM are well known for their capacity to bioaccumulate toxic elements and to alleviate biotic and abiotic stress (e.g., salinity stress) in their hosts. However, the mechanisms underlying AMF involvement in the halophytism of *A. aureum* and the structures where NaCl accumulates remain unknown. This study shows that *A. aureum* forms AM in margins of natural thermal ponds in Neotropical wetlands. All mature sporophytes were colonised by AMF, with high percentages for root length (ca. 57%), arbuscules (23), and hyphae (25) and low values for vesicles (2%). In *A. aureum*–AMF symbiosis, NaCl accumulated in AMF vesicles, and CaSO_4_ precipitated in colonised roots. Therefore, AM can contribute to the halophytic nature of this fern, allowing it to thrive in saline and thermal environments by capturing NaCl from fern tissues, compartmentalising it inside its vesicles, and precipitating CaSO_4_.

## 1. Introduction

Salt surfeit within a plant has detrimental effects on vital processes, such as photosynthesis, protein synthesis, and lipid metabolism. Plant responses to salt stress are greatly complex, involving anatomical, morphological, physiological, biochemical, and molecular mechanisms of salt tolerance. Salinity tolerance differs greatly among plants. Some plants are negatively affected by the presence of excessive minerals; other salt-tolerant plants, such as halophytes, can survive or even thrive on this [1,2,3,4,5]. These plant species grow at a concentration ≥ 200 mM of sodium chloride (NaCl), tolerating or even demanding increased concentrations from the water they absorb [2,6]. In terms of tolerance to and demand for sodium salts, halophytes are classified as obligate, or true, and facultative [7,8].

Halophytism in pteridophytes is notably rare [9]; furthermore, ferns do not grow at sites where salinity exceeds fifty parts per thousand and are absent in arid coastlines with a high soil salinity. The most widely known halophytic fern is the genus *Acrostichum*, named “the mangrove fern”, which has three species that are exceptionally tolerant to salt stress [10,11]: *A. danaeifolium*, *A. speciosum*, and *A. aureum*. Their rhizomes and adventitious roots often grow in flooded soil, although they are never completely submersed. *A. danaeifolium* is restricted to the Neotropics and is the least salt-tolerant [9,12]; *A. speciosum*, from the Paleotropics, is considered the only obligate halophytic fern species [11], and the pantropical species *A. aureum* is halophytic [10,11]. *A. danaeifolium* and *A. aureum* are distributed in Colombia [13], where *A. aureum* proliferates in compacted, saline, and arid alkaline soils [14].

Arbuscular mycorrhizal fungi (AMF) are ubiquitous fungal symbionts belonging to the phylum Glomeromycota [15]. AMF form arbuscular mycorrhiza (AM), an obligate mutualistic symbiosis with the roots and absorption organs of ca. 80% species of both higher- and lower-land plants, including ferns [16,17]. AMF receive carbon compounds from the host; in exchange, they provide plants with limiting nutrients, such as nitrogen and phosphorus, and contribute to the host’s mineral and water access and protection against biotic and abiotic stresses, including pathogens, droughts, heat, and salinity [18]. The exchange of nutrients between symbionts takes place through the cellular interface between the plant and fungus in the colonised host roots. Soil AMF spores colonise roots through hyphae. Hyphae grow inter- and intracellularly in cortical host parenchyma. They branch, forming arbuscules, i.e., fine dichotomously branched hyphae in the host cell; arbuscules are interchanging fungal structures where the bidirectional plant–AMF exchange occurs [19]. Hyphae also develop storage structures: the vesicles. Moreover, AMF hyphae inhabit the soil in a zone that extends beyond the rhizosphere depletion zone, where they capture, hold, and transport nutrients and water to the host [18]. Furthermore, plants have different degrees of dependence on their AM symbiosis, with ferns being obligate, facultative, or non-mycorrhizal [20,21].

AMF are an important group of rhizosphere soil microorganisms that have spread to nearly every ecosystem worldwide, including saline environments [22,23,24,25,26,27,28]. Saline habitats comprise approximately 7% of the global land surface [29,30,31], harbouring similar plant communities. In these plant communities, the zonal distribution of species depends on the soil salt concentration, which, together with biotic factors such as associated fungi, seems to be the driver of plant competitiveness. In saline environments, plant communities include several families of angiosperms considered non-facultative or facultative mycorrhizal plants colonised by glomalean fungi [17,18]. Furthermore, saline soils are often waterlogged and compacted, with increasing salt concentrations; these characteristics reduce AMF spore germination and, therefore, hyphal growth and root colonisation success [32,33]. Although high salt and water content in soils is unfavourable to AMF growth, there are records of AMF colonisation in halophytes [22,23,24,28]. While halophytes are considered to have a low or no ability to associate with AMF [17], some halophytes have been found to be associated, forming AM; for instance, the halophytic fern *Acrostichum aureum* was found to be colonised by AMF in mangroves in China and India [34,35].

It is well known that plants have several anatomo-morphological, biochemical, and functional tools to cope with salt stress [1,7]. Furthermore, AMF–plant associations were proposed as one of a plant’s mechanisms to alleviate and mitigate salinity stress [6,36,37,38,39] because AMF improves plant tolerance to abiotic environmental stresses, including salinity. Although saline environments seem to negatively affect AMF, the positive effects of colonisation on AM plants under salt stress conditions have been reported, such as greater host growth and performance through improved host plant nutrition, a higher K^+^/Na^+^ ratio in plant tissues, and better osmotic adjustment through the accumulation of compatible solutes such as proline, glycine, betaine, or soluble sugars and proteins [6,40,41,42,43]. In host plants under salt stress, AMF also improve the photosynthetic rate as well as water use efficiency and uptake [30,44], stimulate the activity of antioxidant enzymes against the reactive oxygen species generated by salinity, and regulate the expression of the plant genes involved in the maintenance of a better water status in plant tissues, such as the biosynthesis of proline, aquaporins, and late embryogenesis abundant proteins with chaperone activity [45]. Furthermore, gene expression patterns suggest a lower salt stress damage in plants associated with AMF than in non-mycorrhizal plants [6,36,37,38,39,46]. The bulk of information on AMF effects and benefits regarding salinity is related to cultivated hosts, mainly in experimental systems, with reports from native environments and ferns being scarce.

Moreover, AMF have been found to be involved in host tolerance to salt compounds under saline conditions in field soils, greenhouse experiments, and in vitro cultures [46,47,48]. Thus, AMF improve plant tolerance through different mechanisms, including the differential capture and bioaccumulation of elements in the inner root cells colonised by intraradical mycelium and vesicles [48] and in extraradical hyphae and spores [47]. Furthermore, AMF colonisation has effects on the genetic expression of ion transporters in the roots of host plants [46]. However, to our knowledge, whether NaCl could be bioaccumulated in the AMF structures colonising the host roots in saline soil remains unknown.

The concept of sustainable development, which was coined in 1980, is currently based on three pillars: economic, social, and environmental sustainability [49,50]. In particular, the biophysical characteristics of the environment [50] are considered the best indicators of environmental sustainability [50,51] and are directly related to green production strategies in terrestrial environments and blue strategies in aquatic ecosystems, especially in marine systems [51]. Thus, the environmental component of sustainable development represents an important challenge, which is to maintain and improve the integral quality conditions of ecosystems and their resources that support life on Earth and in open water ecosystems, coastal, and wetland areas [52]. Coastal ecosystems, such as mangroves, salt marshes, and wetlands, provide benefits to humans, including food, fishing, recreational, and intangible resources, as key components of economic and social development. These saline ecosystems also provide regulating ecosystem services, like protection from storms, flooding, and erosion, with a strong effect in less developed countries and island states [52]. Furthermore, environmental pressure (climate change, exotic species invasion, overexploitation of fish resources) is stronger in coastal systems in developing countries and island states than in developed countries. Thus, the management and conservation of costal ecosystems and their resources in developing countries are included in the SDG (Sustainable Development Goals) 2030 Agenda [52].

Growth-promoting microorganisms such as AMF are considered tools for environmental sustainability [53,54,55]. In particular, AMF are capable of improving a plant’s nutrition, health, and osmotic response to soil salts, as well as its detoxification through the bioaccumulation of heavy metals. All these functions also contribute to their involvement in the bioremediation of soils [53] and to environmental sustainability through coastal system restoration [54] and coastal wetland conservation [55].

In the Magdalena Department of Colombia, the Córdoba River basin and its wetland coastal zone are considered among the most important water sources. Indeed, the water from this system is used in agriculture, fishing, tourism, and human consumption in the locality of Ciénaga and its surrounding area. Fertilisers and pesticides from agricultural activities are dumped into the basin, in addition to human and animal faeces, with negative effects on water quality, and another agricultural effect is soil salinisation [56]. The halophytic fern *Acrostichum aureum* L. and its mycorrhizal interaction are potential tools for the bioremediation of these brackish wetland areas. Studying this fern–AMF association can provide basic information for its use in the biocapture of salts, heavy metals, and other pollutants generated by agriculture and other anthropogenic activities.

Our aims were to determine if the mangrove fern *A. aureum* is associated with AMF in the coastal wetlands of Neotropical South America, to analyse NaCl salt bioaccumulation in the AMF structures in the roots growing in these saline ecosystems, and to discuss symbiotic NaCl bioaccumulation as a biological mechanism used to cope with the negative effects of salt stress. Our results may help to promote environmental sustainability.

## 2. Materials and Methods

**Figure 1:**
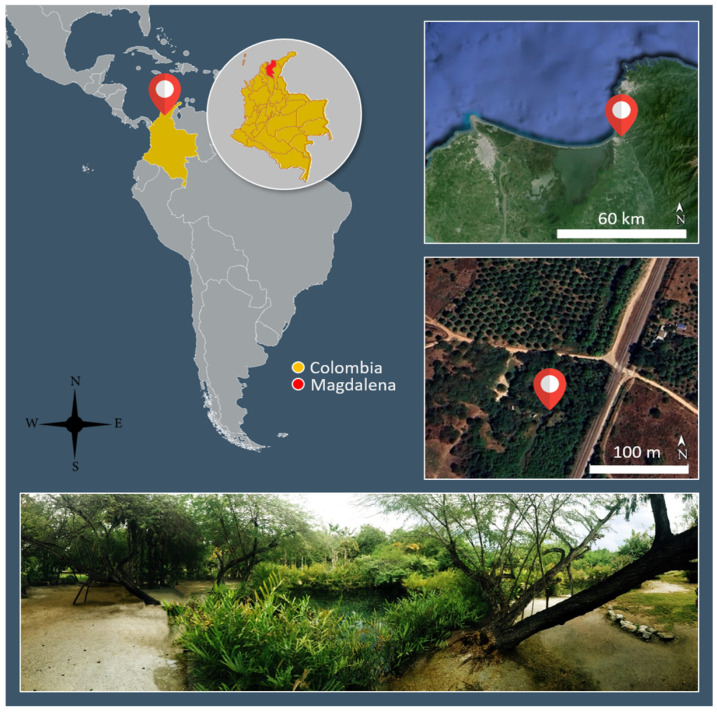
Sampling site in El Volcán thermal ponds, near the Córdoba River mouth in the Caribbean Sea, Magdalena Department, Colombia.

*Acrostichum aureum* L. (Pteridaceae, Polypodales) [57] is distributed in the Caribbean lowlands and in Pacific coastal areas in Colombia, within an elevation range of 0–950 m a.s.l. Samples were taken from El Volcán thermal pond margins, where soil is marshy or flooded; the ponds were located 5 km away from the Córdoba River mouth in the Caribbean Sea, Magdalena Department, Colombia (Figure 1), in March 2023. The sampling area is located at 11.0200278 latitude and 74.2074667 longitude, measured by the data system World Geodetic System 1984 (WGS84) (uncertainty: 1 m; precision: 0.0000001). This area is characterised by riparian vegetation of Fabaceae (*Prosopis juliflora*, *Caesalpinia coriaria*) and Arecaceae (*Attalea butyracea* and *Chamaedorea pinnatifrons*).

Samples of underground rhizomes with adventitious roots were collected from the sporophytes of seven *A. aureum* individuals growing on the margins of thermal ponds located in El Volcán (Figure 1) and stored in plastic bags that were refrigerated at 4 °C until processing. In the laboratory, the fine rhizomes of each sporophyte were washed, cleared, and stained [58]. The finest adventitious roots of the rhizomes of each sporophyte were cut into segments of approximately 1 cm and mounted on semipermanent slides with a 1:3 *v*/*v* water–glycerol solution. Root colonisation by AMF was quantified using an optical microscope at 400 magnification, following the method of McGonigle et al. [59]. For each sporophyte, root segments were mounted on two to three slides, and 180 to 300 intersections were quantified. The colonisation percentage of root (%RL) and each AMF structure (hypha, arbuscule, and vesicle) was statistically analysed with the Kruskal–Wallis test, at α ≤ 0.05, using Infostat software (https://www.infostat.com.ar/, accessed on 17 November 2024). Root slides were observed under a microscope, and the AMF structures colonising roots were microphotographed using a Zeiss AXIO LAB A1 (Carl Zeiss Stiftung, Oberkochen, Germany).

Micrographs from scanning electron microscopy (SEM) were obtained with the Zeiss LEO 1450VP microscope (Zeiss-LEO Electron Microscopy Ltd., Cambridge, UK) at the Laboratory of Electron Microscopy and Microanalysis (LABMEM) at the National University of San Luis (UNSL), Argentina. The chemical composition of the samples was analysed by means of energy-dispersive X-ray spectrometry (EDX) with the EDAX Genesis 2000 spectrometer (EDAX Inc., Mahwah, NJ, USA), along with SEM (SEM-EDX). Samples were mounted on double-sided carbon adhesive tape on aluminium stubs, carbon-coated, and observed at 15 KeV. In addition, vesicles were detached from roots by microdissection under a binocular stereomicroscope (Nikon 7200, Nikon Inc., Melville, NY, USA) at 500 magnification for further observation with SEM and microanalysis by EDX.

The pH (https://www.standardmethods.org/doi/10.2105/SMWW.2882.082, accessed on 9 December 2024), salinity, and conductivity [60] from water and sediments were analysed using Standard Methods Online (https://www.standardmethods.org/doi/book/10.2105/SMWW.2882, accessed on 9 December 2024) at the Laboratorio de Calidad Ambiental Marina (LABCAM) at the Instituto de Investigaciones Marinas y Costeras (INVEMAR, Santa Marta, Colombia). Furthermore, rhizosphere soil was analysed using SEM-EDX for soil element composition. The soil samples were compacted into flat pellets in hollow aluminium stubs and carbon-coated; then, soil pellets were observed at 15 KeV. The carbon layer used to coat the fungal and soil samples was 20 nm thick, and this was carried out by the carbon evaporation method using an SPI coater (Structure Probe Inc., West Chester, PA, USA).

## 3. Results

### 3.1. Chemical Analysis of Thermal Water, Sediments, and Rhizospheric Soil

Thermal water (Table 1) from ponds where *A. aureum* ferns grow was characterised by an acid pH; the presence of nitrates and orthophosphates; a high level of silicates, Zn, and Mn; a high content of Na, K, and Ca; and an electrical conductivity of 3.08 mS/cm. Furthermore, the pond sediments (Table 2) were muddy and presented ammonia, organic matter (OM), Cu, Zn, Fe, Mn, and concentrations of Na and K. In the rhizospheric soil of *A. aureum*, the EDX analysis for elemental composition showed the presence of C, O, Na, Cl, K, Ca, Mg, Fe, Al, Si, and S (Table 3).

### 3.2. Association of Acrostichum aureum with AMF

**Figure 2:**
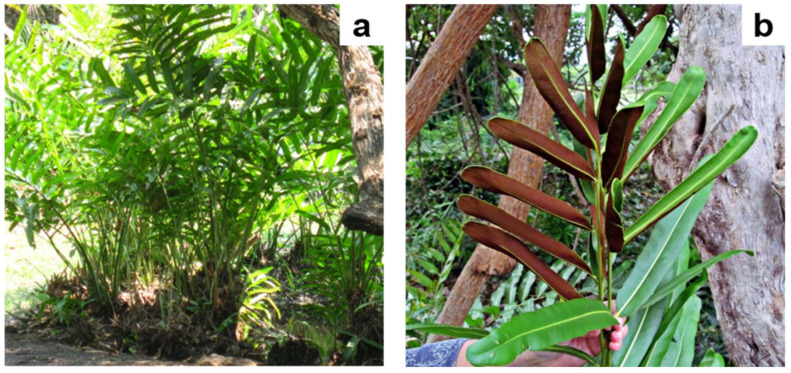
*Acrostichum aureum* in the El Volcán thermal ponds, Córdoba River, Colombia. (**a**) Fern sporophytes growing on the margin. (**b**) Frond of *A. aureum* exhibiting fertile and non-fertile pinnae. Photo credits: (**a**), María A. Negritto; (**b**), Samuel Núñez.

**Figure 3:**
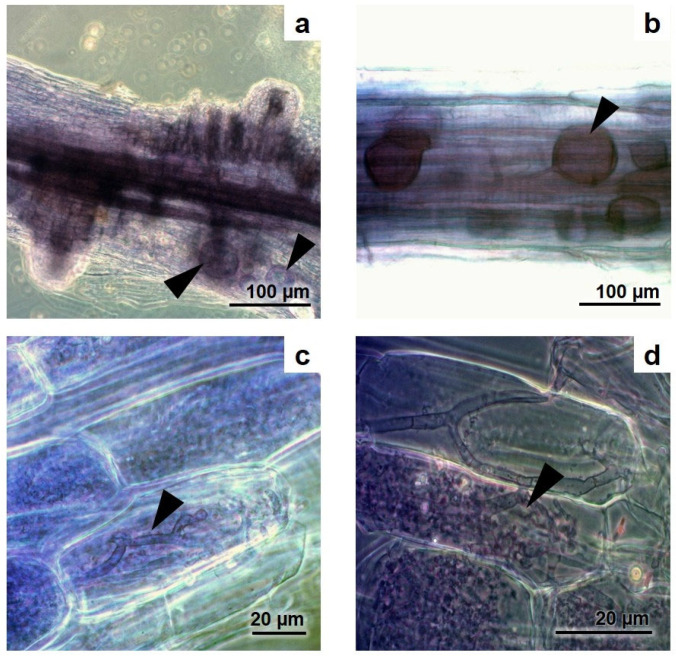
Colonisation by AMF in fine roots of *Acrostichum aureum*. (**a**) General view of a fern root colonised by AMF; (**b**) AMF vesicles; (**c**) AMF hyphal coils and arbuscules; (**d**) AMF arbuscules. Arrowheads indicate illustrated structures. Photo credit: Mónica A. Lugo.

The roots and rhizomes of *Acrostichum aureum* sporophytes growing on the margins of the thermal ponds (Figure 2) were colonised by AMF, which exhibited the characteristic structures in the host roots (Figure 3).

### 3.3. AMF Colonisation of Roots of Acrostichum aureum

The roots of all mature *A. aureum* sporophytes were colonised by AMF; the percentage of colonisation was highest for total root length (RL), followed by the hyphae (HC), arbuscules (AC), and vesicles (VC) (Table 4).

The percentage of total root colonisation and AMF structures (arbuscules, vesicles, and hyphae) showed no significant differences among mature individuals of *A. aureum* in the thermal ponds studied.

### 3.4. Salt Crystals of NaCl in Acrostichum aureum Roots and in AMF Intraradical Structures

**Figure 4:**
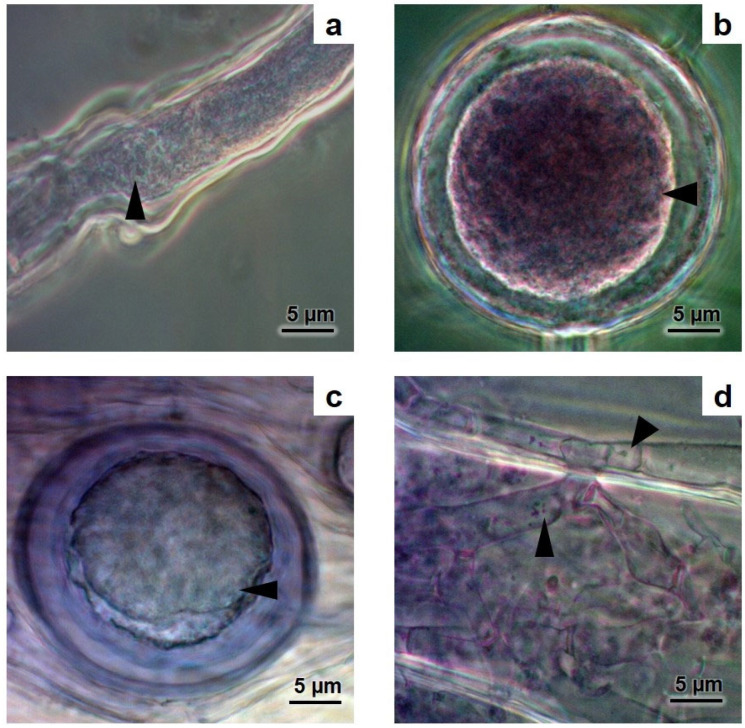
AMF structures outside and inside fine roots of *Acrostichum aureum* with dense cytoplasmic content (arrowhead). (**a**) AMF hypha; (**b**) AMF spores outside roots; (**c**) AMF vesicles; (**d**) AMF arbuscules inside roots. Photo credit: Mónica A. Lugo.

**Figure 5:**
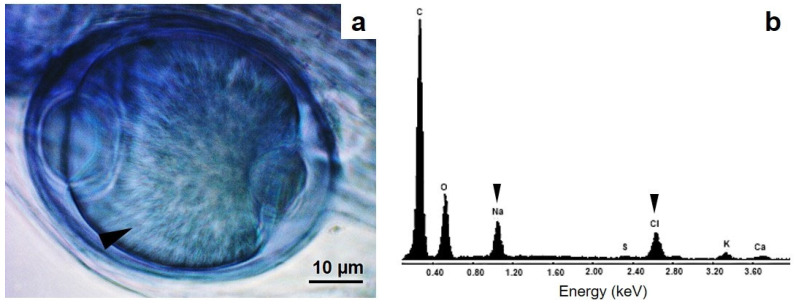
Salt crystals inside AMF vesicles. (**a**) Vesicles of AMF with NaCl crystals (indicated by the arrowhead) observed under optical microscope. (**b**) The EDX analysis of the crystals in the vesicles showed the elemental composition of NaCl. NaCl crystals and each salt element are indicated by arrowheads. Photo credit: Mónica A. Lugo.

The extraradical structures of hyphae and spores and the intraradical arbuscules and vesicles of the AMF associated with *A. aureum* showed dense cytoplasmic content (Figure 4). Salt crystals were also observed inside the vesicles (Figure 5a).

**Figure 6:**
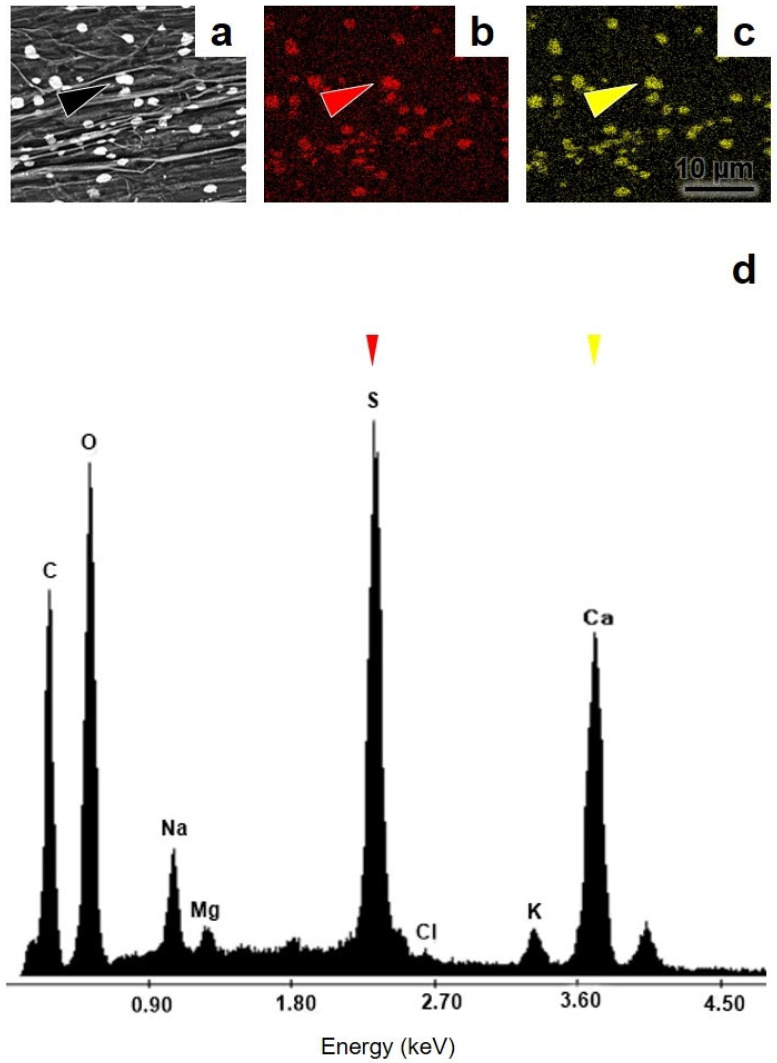
Salts accumulated inside roots of *Acrostichum aureum* analysed by SEM-EDX. (**a**) SEM image of amorphous crystals obtained with the backscattered electron detector (black arrowhead). (**b**) X-ray mapping indicating the distribution of S in an *A. aureum* root (red arrowhead). (**c**) X-ray mapping indicating the distribution of Ca in the root (yellow arrowhead). (**d**) EDX spectrum of amorphous crystals, showing their CaSO_4_ composition (red and yellow arrowheads).

The EDX analysis showed that the crystals present in the vesicles are composed mainly of NaCl (Figure 5b), with very low proportions of K, Ca, and S. The other elements inside the vesicles were C, O, S, K, and Ca (Table 5). In addition, the amorphous crystals accumulated inside the roots were composed of CaSO_4_ (Figure 6a–c). Other elements such as K and Na accounted for 1% of the total elements around the crystals, whereas Cl and Mg were also detected as traces lower than 1% (Figure 6d).

## 4. Discussion

In the Colombian wetlands of Córdoba River, we observed that the roots and rhizomes of *Acrostichum aureum* sporophytes growing on the margins of the thermal ponds were colonised by AMF, which formed their characteristic structures in the host roots. In Asian mangroves, *A. aureum* root colonisation ranged between high and low values, with colonisation percentages varying with the flooding level [35], and roots were found to form arbuscules and vesicles in Goa, India [27], and China [34,35]. In contrast, *A. aureum* was considered non-colonised in other Indian mangroves [61,62,63]. As far as we know, this is the first report of AMF colonisation in roots and rhizomes of *A. aureum* in plant communities associated with Colombian mangroves and thermal ponds in a Neotropical area. In this Neotropical wetland, the total AMF colonisation and percentage of arbuscules were high. Arbuscules are structures for nutrient exchange between the host and fungus [18,19]; therefore, these high values suggest that the symbiotic interaction between *A. aureum* and AMF in the thermal ponds was functioning actively as mutualistic.

In this work, we found AMF colonisation in the roots of *A. aureum*, which is in line with the results of *A. aureum* growing in some Asian mangroves; AMF colonisation was also recorded in the fern rhizomes. We also found AMF colonisation in thermal ponds in the Colombian Neotropical area, which is not a mangrove but has been a wetland in the past. Moreover, the water temperature (47 °C) of these thermal ponds, where *A. aureum* roots and rhizomes were colonised, is the highest value ever detected for this fern, with previous reports indicating 29–30 °C [61]. Thus, the occurrence of A. *aureum* in Colombian thermal ponds and its mycorrhizal association widen its known distribution; in addition, the growth temperature detected is the highest value recorded for this species so far.

In addition, in the Volcán thermal ponds, extraradical structures such as hyphae and spores, as well as the intraradical arbuscules and vesicles of the AMF associated with *A. aureum*, showed dense cytoplasmic content and salt crystals, which were found within the vesicles. In previous reports of AMF colonisation and structures [27,34,35,61], these dense contents inside AMF structures were not reported for *A. aureum*. It is known that AMF can improve plant tolerance to salt stress through different mechanisms, including the bioaccumulation of compatible solutes and elements [63] in the inner root cells colonised by intraradical mycelium and vesicles [48] and extraradical hyphae and spores [47]. As far as we know, this is the first report about the dense cytoplasmic content in AMF associated with the halophytic fern *A. aureum* growing in the Neotropics and thermal ponds.

The EDX crystal analysis showed that the crystals in the vesicles were composed of NaCl inside the cortical root cells of *A. aureum* growing in the thermal ponds. Olsson et al. [48] analysed the roots of leak growing under greenhouse conditions using PIXE in combination with STIM and found that P was the most common element in the vesicles, followed by K and Ca. Furthermore, the authors found Zn, Si, Al, S, Mn, Cu, and Cl inside the vesicles. On the other hand, Hammer et al. [47] studied *Acacia cyanophylla*–AMF association in a field experiment using bioassays under high salinity conditions; the authors found significantly high levels of Ca, Cl, Mg, Fe, Si, and K, but not Na, which was present at a much lower concentration than its Cl counter-ion in extraradical hyphae and spores. Although in the field samples, Na and Cl were present inside extraradical hyphae and spores, Hammer et al. [47] concluded that extraradical AMF hyphae and spores are selective in capturing these elements and that they are able to avoid Na incorporation in the plant when the ratios of K/Na and Ca/Na are considered in field and in vitro culture experimental approaches [47]. However, we also found NaCl crystals inside the root vesicles of *A. aureum* with the same Na and Cl levels as those of field-sampled roots and rhizomes; indeed, this is the first report of NaCl inside vesicles of AMF colonising the roots of the halophytic fern *A. aureum*. Furthermore, the presence of NaCl salt crystals in the vesicles suggests that the role of AMF in colonising the roots of this halophyte fern is that of bioaccumulating salt inside the fungus instead of avoiding Na and Cl accumulation, thereby providing the plant with another mechanism for avoiding salt incorporation.

Medina et al. [64] concluded that the distribution of *A. aureum* at three mangrove sites with different soil salinities “is not due to lack of salt resistance in the sporophyte”. In these mangrove areas in Puerto Rico, the Cl^−^/Na^+^ ratio in the cell sap of *A. aureum* was twice the values obtained for the other mangrove species. Furthermore, *A. aureum* has a K^+^/Na^+^ ratio of 2.6, whereas typical mangrove species have a ratio of 0.5. The authors attributed these results to the presence of ion selectivity, probably of Na^+^, at the root level. Furthermore, Na^+^ exclusion from the roots and AMF structures was found to be a mechanism for avoiding salinity in *Acacia cyanophylla* (a halophyte host in the desert of Turkey); this plant benefited from AMF since it prevented Na^+^ capture while increasing K^+^ and Ca^2+^ capture [47]. In contrast to this mechanism, the NaCl crystals present inside the AMF vesicles of colonised *A. aureum* roots could be functioning as differential ion selectors in these saline environments, with this representing a different mechanism for overcoming environmental salinity in this halophytic fern. Therefore, this result may explain ion selectivity at the root level [64] inside the roots of this halophytic fern through AMF colonisation and NaCl bioaccumulation.

In Córdoba River thermal ponds, *A. aureum* presented gypsum salt accumulation inside roots, which was mainly composed of CaSO_4_ crystals, with lower proportions of Na and K, and trace elements such as Cl and Mg. It is known that the Ca^2+^/Na^+^ ratio is an important parameter for measuring salt stress in plants. Under salt stress conditions, Ca^2+^ absorption is interfered with by the elevated rhizospheric Na^+^ concentration, and Ca^2+^ is replaced at the cell wall and plasma membrane levels. Consequently, Ca^2+^ translocation and the Ca^2+^/Na^+^ ratio are reduced in plants under salt stresses. Furthermore, a low Ca^2+^/Na^+^ ratio causes a decrease in hydraulic conductivity and cell turgor, along with interruptions in Ca^2+^ signalling. Although the presence of Mg^2+^ affects Ca^2+^ uptake, mycorrhizal plants accumulate more Mg^2+^ than non-mycorrhizal plants and show an advantageous Ca^2+^/Na^+^ ratio due to their Ca^2+^ uptake improvement under salt stress conditions [45]. Although the mechanism involved in these processes remains unknown, mycorrhizal colonisation was found to help the host plant overcome the deficiencies generated by Na^+^, Ca^2+^, and K^+^ while maintaining beneficial K^+^/Na^+^, Ca^2+^/Na^+^, and Ca^2+^/Mg^2+^ ratios in its tissues [63].

Furthermore, Ca^2+^ functions as a signal for root development and adaptation to optimise the uptake of phosphate (Pi), an important plant growth-limiting factor. However, Ca^2+^ has to be controlled due to its high toxicity to Pi metabolism [65]. Moreover, depending on the soil environment pH, Pi precipitates in the presence of Ca^2+^, Mg^2+^, and Zn^2+^ cations, becoming unavailable to plants [31,45] and generating salt-induced plant Pi deficiency [45]. Thus, inside the *A. aureum* roots, Ca was retained as a CaSO_4_ salt, forming amorphous crystals that made Pi absorption possible for the plant. Moreover, Ca^2+^ formed salts with sulphates and contributed to the removal of SO^4−^ ions from root media, avoiding H_2_SO_4_ formation and the consequent acidification of the thermal water and rhizospheric soil that *A. aureum* inhabits. Therefore, the CaSO_4_ amorphous crystals in *A. aureum* roots could have a dual alleviation function under saline stress: contributing to Pi absorption and balancing or buffering pH in the roots of this fern. Our results failed to explain whether gypsum salt is formed by one of the partners or synergistically through *A. aureum*–AMF arbuscular mycorrhizal symbiosis. However, it is clear that the presence of CaSO_4_ within *A. aureum* roots is a beneficial mechanism for this fern to thrive under saline stress.

Several studies suggest that sulphur-bioaccumulator plants (thiophores, gypsophiles, and gypsovags) produce mainly foliar CaSO_4_ salts [66]. The presence of root CaSO_4_ was reported only for *Acacia ancistrocarpa* [67]. Gypsum salt bioaccumulation has been considered a biomineralogical tolerance mechanism against sulphate salinity and soluble calcium excess in plants capable of settling in abiotically stressed soils, such as those in deserts, coastal, and riparian zones, where the presence of soil gypsum does not necessarily indicate that the plant is a bioaccumulator of this mineral [67,68]. The growth of gypsum-forming plants was also limited by Pi unavailability, caused by the scarce soluble calcium-phosphate formation in the soils. However, Robson et al. [66] stated that the capacity for gypsum biomineralization did not confer thiophores with the tolerance needed to overcome Pi deficiency per se. In this study, surprisingly, gypsum crystals, which are usually stored in the leaves and shoots of most gypsophiles and thiophores, were detected within *A. aureum* roots. In this mycorrhizal halophytic fern, these CaSO_4_ crystals may increase Pi availability and capture from saline soils, cooperating with the *A. aureum*–AMF association, which efficiently improves the host’s Pi nutrition [18]. Furthermore, as far as we know, this study is the first to describe the presence of gypsum salts within fern roots. Experimental studies are necessary to confirm whether *A. aureum* is a thiophore fern and to understand the process of amorphous crystal production and its role in osmoregulation mechanisms.

The results of this study show that the halophytic pioneer fern *A. aureum*, characteristic of the vegetation in pantropical mangroves and swamps, can also grow in thermal water from Neotropical Colombian springs. Furthermore, the bioaccumulation of NaCl crystals was observed in AMF vesicles within *A. aureum* roots, as well as amorphous CaSO_4_ crystals, suggesting that this species is a thiophore. Therefore, in this fern, mycorrhizal colonisation facilitates a wide range of mechanisms, either inherent to the species or provided by its AM symbionts. These mechanisms allow the plant to thrive in environments with high stress levels and extreme environmental conditions, such as saline and flooded soils and thermal waters, enabling the availability of Pi in the rhizosphere, its capture, and its later translocation, consequently allowing *A. aureum* to be a “conqueror” fern. The capacity for an *A. aureum*–AMF association for salt bioaccumulation in these coastal environments constitutes an important attribute for the involvement of this fern in environmental sustainability [49,50,51,52] in similar wetland saline systems.

In mangroves, marshes, swamps, and wetlands in Africa and Asia, *A. aureum* is widely used as an edible plant. It also has various ethnobotanical uses in traditional medicine as an analgesic, antibacterial, antidiarrheal, anthelmintic, anti-inflammatory, antioxidant, and healing agent. This mangrove fern also produces numerous advantageous phytochemical compounds (e.g., kaempferol, di-(2-methylheptyl) phthalate, β-sitosterol, (2S,3S)-sulphated pterosin C, (+)-pinoresinol-4-O-sulphate, lupeol, α-amyrin, and phytol). Furthermore, extracts of *A. aureum* have been used for the treatment of cancer, diabetes, ulcers, and viral diseases [69,70]. On the American continent, *A. aureum* is also considered an edible plant and has been widely consumed in Mesoamerica since the times of the Maya people [71] up until today [72]. In North America, *A. aureum* is used as a diuretic and expectorant [73], as well as in Mesoamerica and South America [72,74]. In addition, *A. aureum* has been recorded in Colombia as providing some different health consumables, such as herbal medicine for constipation, fever, malaria, and rheumatism, among other human diseases, and as an expectorant, fodder, and bed for livestock and for ornamental use [72,73,74].

For more than 2500 years, humans have used plants for medicinal purposes. Currently, they are used directly as a source of herbal medicine to cure diseases in most underdeveloped countries, particularly in tropical and subtropical areas, and indirectly, they are used as providers of active compounds and chemical substances in the pharmaceutical industry for the production of commercially available drugs [75]. Medicinal plants are being threatened with extinction due to habitat loss and pollution. At the same time, the increase in their demand, the concomitant increase in their production, and the standardisation required for their commercialization have contributed to a marked increase in their intensive cultivation [76,77]. Likewise, the large-scale production of medicinal plants implies a detriment to their medicinal qualities, such as a decrease in the contents of their active ingredients and their metabolic activity [78,79].

Symbiotic interactions between medicinal plants and endophyte root fungi such as AMF [79,80] have received special interest worldwide due to the benefits that these fungal symbionts provide to their host plants, improving plant nutrition and increasing plant productivity and resistance to pathogens and drought [18]. In particular, AMF and AM are essential for medicinal plant cultivation due to AMF’s primary role in increasing active compounds, medicinal secondary metabolites, and the metabolic activity of the host, with a consequent increase in the pharmaceutical quality of mycorrhizal plants [81,82]. Furthermore, *A. aureum* has shown promising potential in the phytoremediation of environmental contaminants like heavy metals since this plant contributes to heavy metal removal from soil, wastewater, and effluents, especially in marine wetlands. In addition, this species is highly tolerant to arsenate toxicity [70]. In this work, we have revealed the bioaccumulation capacity of NaCl salts from the soil and water of *A. aureum* through its AMF-associated symbionts. Thus, the *A aureum*–AMF association in tropical saline areas such as Córdoba River thermal ponds suggests the synergistic potential of this fern for the economic and social pillars of sustainability [49,50] in this stressed coastal environment, whose sustainability is under huge threat [52].

## 5. Conclusions

Mature sporophytes of *A. aureum* occurring in Colombian thermal ponds, an anthropogenically stressed coastal environment of the Caribbean basin, were associated with AMF, showing high percentages for root length, arbuscules, and hyphal colonisation. AMF root colonisation in *A. aureum* could enhance phytoremediation by promoting the growth of hyperaccumulating, halophytic, and salt-tolerant plants capable of bioameliorating saline soils. Additional experimental studies are necessary to understand the mechanisms involved in salt bioaccumulation in AMF structures, NaCl and CaSO_4_ osmoregulation, and fern–AMF symbiosis and nutrition processes. *A. aureum*–AMF symbiosis would also be useful as a green tool applied to increase the environmental sustainability of tropical wetlands. Furthermore, its numerous ethnobotanical uses, medicinal properties, and pharmacological substances, which are applicable to human health, make this fern an excellent renewable natural resource related to the other two pillars of sustainable development: economic and social sustainability. The *A. aureum*–AMF association can be seen as a key holobiont in the sustainable development of seriously threatened coastal environments, especially in developing countries in the Neotropics. Future works on this fern–AMF symbiosis should address the seasonality of AMF sporulation and AM colonisation, since AMF symbiosis varies seasonally and the fluctuations in precipitation between the wet and dry seasons in the tropics can modify salinity. Bioassays should also be conducted to analyse symbiosis in non-colonised controls, and inoculation with known native AMF taxa should be performed to obtain more precise data on this interaction.

## Figures and Tables

**Table 1:** Chemical analysis of the thermal water where roots and rhizomes of *Acrostichum aureum* grow in the study area.

Physico-Chemical Variables	Values
pH	5.45
Electrical Conductivity (Ms/cm)	3.08
N-NO_2_ (µg/L)	<LD *
N-NO_3_ (µg/L)	2.5
N-NH_4_ (µg/L)	<LD *
P-PO_4_ (µg/L)	2.4
Si-SiO_4_ (µg/L)	13916.1
Pb (µg/L)	<LD *
Cd (µg/L	<LD *
Cr (µg/L)	<LD *
Cu (µg/L)	<LD *
Zn (µg/L)	13.4
Ni (µg/L)	<LD *
Fe (µg/L)	<LD *
Mn (mg/L)	0.5
Na (mg/L)	240.3
K (mg/L)	4.6
Mg (mg/L)	<LD *
Ca (mg/L)	157.5

* LD: lower detection value than the methodological detection power.

**Table 2:** Physico-chemical analysis of thermal sediments where roots and rhizomes of *Acrostichum aureum* grow.

Physico-Chemical Features’ Texture	Values
2 mm	3.3%
1 mm	11.5%
0.5 mm	16.9%
250 µm	16.3%
125 µm	12.3%
63 µm	7.1%
≤63 µm	32.7%
**Chemical Composition**	**Values**
OM ox (mg/g)	179.5
OM vol (mg/g)	266.3
Humidity (% H_2_O/wet weight)	60.0
N-NO_3_ (µg/g)	<LD *
N-NH_4_ (µg/g)	23.4
P-PO_4_ (µg/g)	<LD *
Cu (µg/g)	7.8
Zn (µg/g)	56.7
Fe (µg/g)	20.4
Mn (mg/g)	248.2
Na (mg/g)	1.8
K (mg/g)	2.1

* LD: lower detection value than the methodological detection power.

**Table 3:** EDX chemical analysis of rhizospheric soil of *Acrostichum aureum* in thermal soils. The values are the mean of four replicates of EDX measurements.

Elements—K	Values * (% Element/Soil Sample Weight)
C K	32.75 ± 2.16
O K	32.18 ± 0.35
Na K	1.75 ± 0.19
Mg K	0.62 ± 0.07
Al K	5.61 ± 0.33
Si K	18.43 ± 1.45
S K	0.41 ± 0.26
Cl K	0.60 ± 0.05
K K	1.05 ± 0.16
Ca K	3.91 ± 0.33
Fe K	2.71 ± 0.47

*—values lower than methodological detection power, with percentages ˂ 1% and main elements > 10%; K = atomic energetic level of each element used as a measuring reference value; data are mean value ± standard deviation.

**Table 4:** Arbuscular mycorrhizal fungi (AMF) colonisation of roots of mature *Acrostichum aureum* sporophytes in thermal soils.

AMF Structures	Percentage of Colonisation
RL	57.93 ± 3.05
HC	25.00 ± 10.64
AC	23.75 ± 24.62
VC	9.29 ± 11.15

RL = total-root-length colonisation; HC = hyphal colonisation; AC = arbuscular colonisation; VC = vesicular colonisation; data are mean ± standard deviation.

**Table 5:** EDX chemical analysis of vesicles of AMF in roots of *Acrostichum aureum* in thermal soils. The values are the means of three replicates of EDX measurements.

Elements—K	Values * (% Element/Soil Sample Weight)
C K	77.13 ± 7.14
O K	15.54 ± 5.31
Na K	2.30 ± 1.45
S K	1.07 ± 0.20
Cl K	2.69 ± 0.64
K K	1.13 ± 0.33
Ca K	0.85 ± 0.15

*—values lower than methodological detection power with percentages ˂ 1% and main elements > 10%; K = atomic energetic level of each element used as a measuring reference value; data are mean value ± standard deviation.

## Data Availability

The original contributions presented in the study are included in the article, further inquiries can be directed to the corresponding author.
